# Istaroxime, a potential anticancer drug in prostate cancer, exerts beneficial functional effects in healthy and diseased human myocardium

**DOI:** 10.18632/oncotarget.17540

**Published:** 2017-04-30

**Authors:** Markus Wallner, Mounir Khafaga, Ewald Kolesnik, Aris Vafiadis, Gerold Schwantzer, Deborah M. Eaton, Pero Curcic, Martin Köstenberger, Igor Knez, Peter P. Rainer, Martin Pichler, Burkert Pieske, Dirk Von Lewinski

**Affiliations:** ^1^ Division of Cardiology, Department of Internal Medicine, Medical University of Graz, 8036 Graz, Austria; ^2^ Cardiovascular Research Center, Lewis Katz School of Medicine, Temple University, Philadelphia, 19140 PA, United States of America; ^3^ Institute for Medical Informatics, Statistics, and Documentation, Medical University of Graz, 8036 Graz, Austria; ^4^ Division of Cardiac Surgery, Department of Surgery, Medical University of Graz, 8036 Graz, Austria; ^5^ Department of Pediatric Cardiology, Medical University of Graz, 8036 Graz, Austria; ^6^ Division of Clinical Oncology, Department of Internal Medicine, Medical University of Graz, 8036 Graz, Austria; ^7^ Department of Internal Medicine and Cardiology, Campus Virchow Klinikum, Charité University Medicine, Berlin, 13353 Berlin, Germany; ^8^ Department of Internal Medicine and Cardiology, German Heart Center, Berlin, 13353 Berlin, Germany

**Keywords:** prostate cancer, hormone therapy, cardiac disease models, heart failure, istaroxime

## Abstract

The current gold standard for prostate cancer treatment is androgen deprivation therapy and antiandrogenic agents. However, adverse cardiovascular events including heart failure can limit therapeutic use. Istaroxime, which combines Na^+^-K^+^-ATPase (NKA) inhibition with sarco/endoplasmic reticulum Ca^2+^-ATPase 2a (SERCA2a) stimulation, has recently shown promising anti-neoplastic effects in prostate cancer (PC) models and may also improve cardiac function. Considering the promising anticancer effects of istaroxime, we aimed to assess its functional effects on human myocardium.

**Results:**

Istaroxime and strophanthidin elicited dose-dependent positive inotropic effects with a decline in developed force at supraphysiological concentrations in human atrial, nonfailing, and failing ventricular (ToF) myocardium. Diastolic force and RT50% did not change after exposure to both drugs. The maximal developed force in our *in-vitro* model of heart failure (ToF) was significantly higher after istaroxime administration. Such a difference did not occur in atrial or nonfailing ventricular trabeculae and was not applicable to the diastolic force.

**Materials and Methods:**

Human atrial and ventricular trabeculae were isolated from nonfailing hearts and hearts of infants with tetralogy of Fallot (ToF), which were used as an *in-vitro* model of heart failure. The samples were electrically stimulated and treated with increasing concentrations of istaroxime and strophanthidin (10 nM–1 μM). Systolic and diastolic force development and relaxation parameters (RT50%) were analyzed.

**Conclusions:**

Combined NKA inhibition/SERCA2a stimulation increases contractility in atrial, nonfailing, and failing myocardium. Considering that heart failure is a potential side effect of current PC treatments, especially in elderly patients, istaroxime might combine beneficial cardiac and anti-cancer properties.

## INTRODUCTION

Prostate cancer (PC) is the most common malignant disease in elderly men in western countries [1]. There has been an increase in the availability of diagnostic tools for PC, such as prostate-specific-antigen-based screening tests, and advancements in therapeutics that have resulted in a decline in the total number of fatal PC rates [2]. Despite the improvements in both diagnosing and treating the disease, it is still associated with moderate mortality rates, partially attributed to stable rates of fatal PC in younger men [3]. A study using the “Surveillance, Epidemiology, and End Results (SEER)” data of the United States found that PC accounts for approximately 19% of all newly diagnosed cases of cancer in men and approximately 8% of all deaths due to cancer in men in 2017 [1]. The study also reported a median age at time of diagnosis of 66 years and a median age at time of death of 80 years, underlining the age-associated nature of this disease [4]. The current gold-standard conservative approach for PC is androgen deprivation therapy, which is used as an adjunctive treatment strategy in localized high-risk PC or as a continuous treatment for metastatic PC patients. In addition, novel antiandrogens (enzalutamide) and androgen biosynthesis inhibitors (abiraterone) are approved in the setting of metastatic PC before and after docetaxel chemotherapy. Furthermore, these agents are currently being investigated in clinical trials for their efficacy in a non-metastatic PC setting [5]. However, increased risk of cardiovascular diseases, such as coronary artery diseases, acute myocardial infarction and heart failure (HF), are reported as limiting side effects and contraindications for some of these drugs. This may significantly limit their therapeutic use [6–10].

Istaroxime, a novel drug originally developed for treating HF, received attention for its potential anti-neoplastic effects. Istaroxime inhibits Na+-K+-ATPase (NKA), like strophanthidin, and exerts additional stimulatory effects on the sarco/endoplasmic reticulum Ca^2+^-ATPase 2a (SERCA2a). It has exhibited lusitropic and inotropic properties in experimental and early clinical studies [11]. Preclinical studies and clinical trials indicate that combining SERCA2a activation and NKA inhibition may increase contractility and facilitate active relaxation, improving systolic as well as diastolic heart function, both of which would be beneficial effects in the treatment of chronic HF [12, 13]. Early data from human studies have shown that istaroxime increases systolic blood pressure, decreases heart rate, decreases pulmonary capillary wedge pressure, and possibly improves diastolic function without increasing myocardial oxygen consumption or causing a significant change in ischemic or arrhythmic events [13–16]. However, larger clinical trials with the purpose of exploring the clinical usage of istaroxime in HF were never performed, thus guideline-based indications do not exist.

Although istaroxime may not make it to market as a HF drug, it continues to show potential as an anti-cancer drug. Istaroxime showed promising anti-neoplastic effects in the cell lines of 9 different types of cancer and a reduction of tumor growth *in vivo* in PC xenograft models. Furthermore, the authors of this study identified a putative crosstalk between the inhibition of NKA and membrane-bound androgen receptor and down-stream signaling [17]. This data fits well with the results of previous studies, which demonstrated that NKA inhibitors have a strong anti-neoplastic potential [18–20]. Of note, HF and PC share risk factors such as age and hyperlipidaemia [21, 22]. Since the cardiovascular complications of current androgen deprivation therapeutics can significantly limit their usage, evaluating the cardiovascular safety profiles of newly developed drugs is essential.

Therefore, we tested the functional effects of istaroxime on human nonfailing atrial and ventricular myocardium and a unique *in-vitro* model of heart failure with diastolic dysfunction. This model is especially valuable considering that impaired relaxation is a common condition in many elderly patients who have HF with preserved ejection fraction. For comparison, we performed experiments with the same conditions using the NKA inhibitor strophanthidin, as previous studies have already determined its functional impact on human myocardium [23].

## RESULTS

### Functional effects of istaroxime and strophanthidin in human atrial myocardium

**Figure 1:**
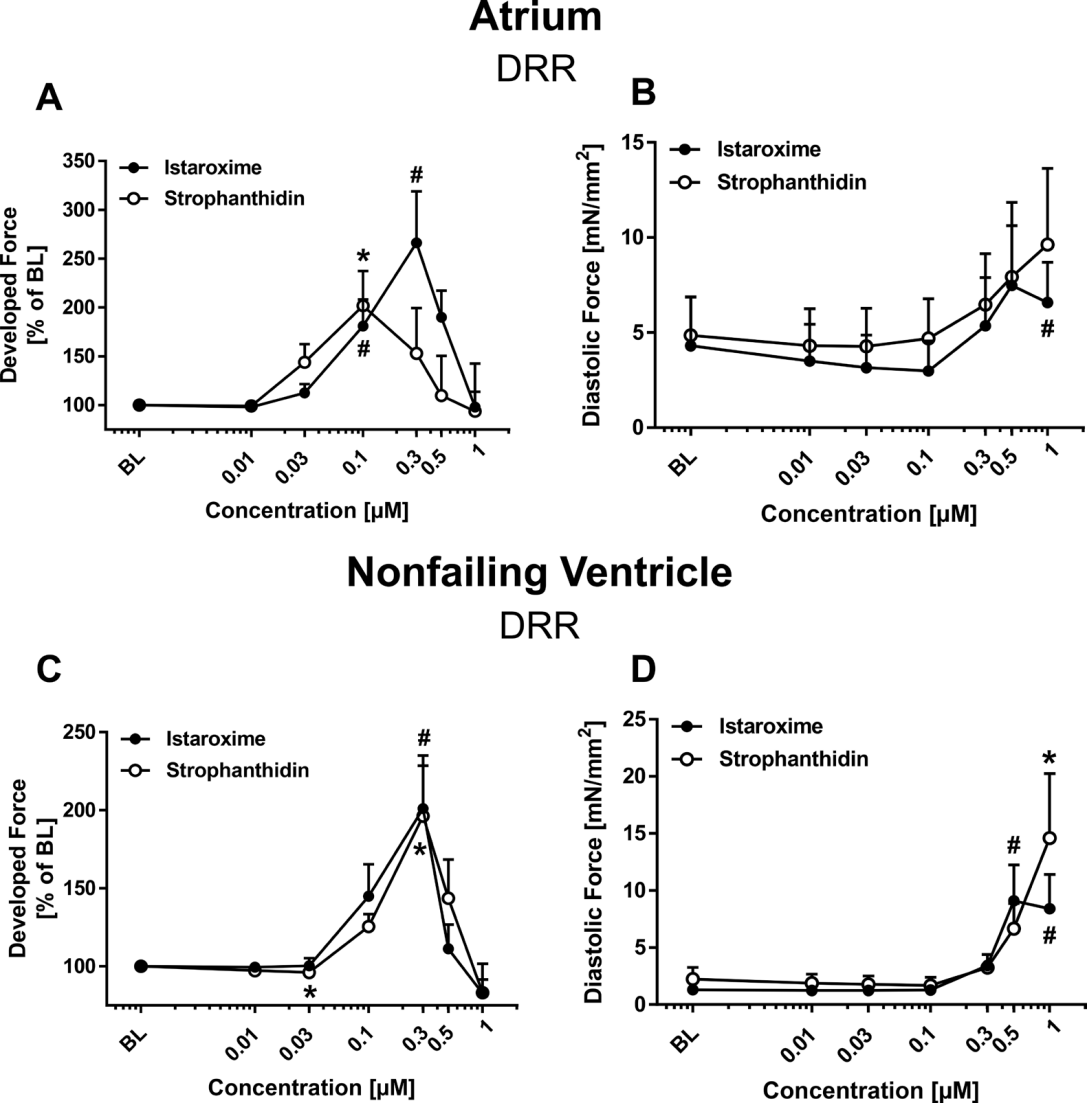
Inotropic effects of istaroxime and strophanthidin on human atrial and nonfailing ventricular myocardium Comparable effects of istaroxime and strophanthidin on developed and diastolic force in human atrial (**A**–**B**) and nonfailing ventricular (**C–D**) myocardium. Data are represented as mean +/− SEM. (A) Dose response relationship (DRR) for strophanthidin (open circles; *n* = 13 trabeculae) and istaroxime (filled circles; *n* = 8 trabeculae). Effect on developed force: *= *p* < 0.05 vs. baseline (strophanthidin); ^#^ = *p* < 0.05 vs. baseline (istaroxime). (*B*) DRR for strophanthidin (open circles) and istaroxime (filled circles) with effect on diastolic force; ^#^ = *p* < 0.05 vs. baseline (istaroxime). (C) DRR for strophanthidin (open circles; *n* = 9 trabeculae) and istaroxime (filled circles; *n* = 9 trabeculae). Effect on developed force: * = *p* < 0.05 vs. baseline (strophanthidin); ^#^= *p* < 0.05 vs. baseline (istaroxime). (D) DRR for strophanthidin (open circles) and istaroxime (filled circles) with effect on diastolic force: ^#^= *p* < 0.05 vs. baseline (istaroxime).

We assessed the effects of NKA inhibition alone (strophanthidin; *n* = 13 trabeculae) versus NKA inhibition combined with SERCA stimulation (istaroxime; *n* = 8 trabeculae) on developed force in human atrial trabeculae. Increasing concentrations of strophanthidin increased the developed force with a maximum effect at 0.1 μM. The developed force declined further as the concentration was increased. Similarly, increasing concentrations of istaroxime (black circles) produced a positive inotropic effect with a maximum effect at 0.3 μM and subsequent force decline at higher concentrations (Figure 1A). Both strophanthidin and istaroxime slightly increased diastolic force compared to baseline in a dose-dependent manner with comparable kinetics of the curves (Figure 1B). Next, we performed force-frequency relationship (FFR) experiments by increasing stimulation frequencies stepwise (0.5–3.0Hz) before and after incubation with either istaroxime or strophantidin. FFR results are shown in the supplement (Supplementary Figure 1A–1D). Similar effects between strophanthidin and istaroxime could be observed. No arrhythmic events were detected for istaroxime or strophanthidin in any of the experiments performed. The RT50%, a relaxation parameter, did not reveal any significant differences between istaroxime and strophanthidin in dose response relationship (DRR) or FFR experiments (Supplementary Figure 3A–3C).

### Functional effects of istaroxime and strophanthidin in human nonfailing ventricular myocardium

Next, we investigated the effects of istaroxime and strophanthidin in isolated ventricular trabeculae from human nonfailing donor hearts. Analogous to our findings in atrial trabeculae, strophanthidin (*n* = 9 trabeculae) and istaroxime (*n* = 9 trabeculae) increased developed force in ventricular trabeculae (Figure 1C) in a dose-dependent and comparable manner. Maximum effect on systolic force was seen at 0.3 μM for both substances. Developed force declined as the concentration was increased up to 1 μM. Figure 1D shows the effect of strophanthidin and istaroxime on diastolic force. Both substances showed a dose-dependent increase in diastolic force compared to baseline with comparable kinetics of the curves. Results from the FFR in ventricular nonfailing human myocardium are presented in the supplement (Supplementary Figure 2A–2D). As observed in the FFR protocol in atrial trabeculae, strophanthidin and istaroxime exerted similar effects and no arrhythmic events could be detected for istaroxime or strophanthidin in any experiments. Consistent with the findings in atrial myocardium, the RT50% was not different between istaroxime and strophanthidin in neither the DRR nor in the FFR experiments (Supplementary Figure 3D–3F).

### Functional effects of istaroxime and strophanthidin in failing ventricular myocardium from patients diagnosed with Tetralogy of Fallot

**Figure 2:**
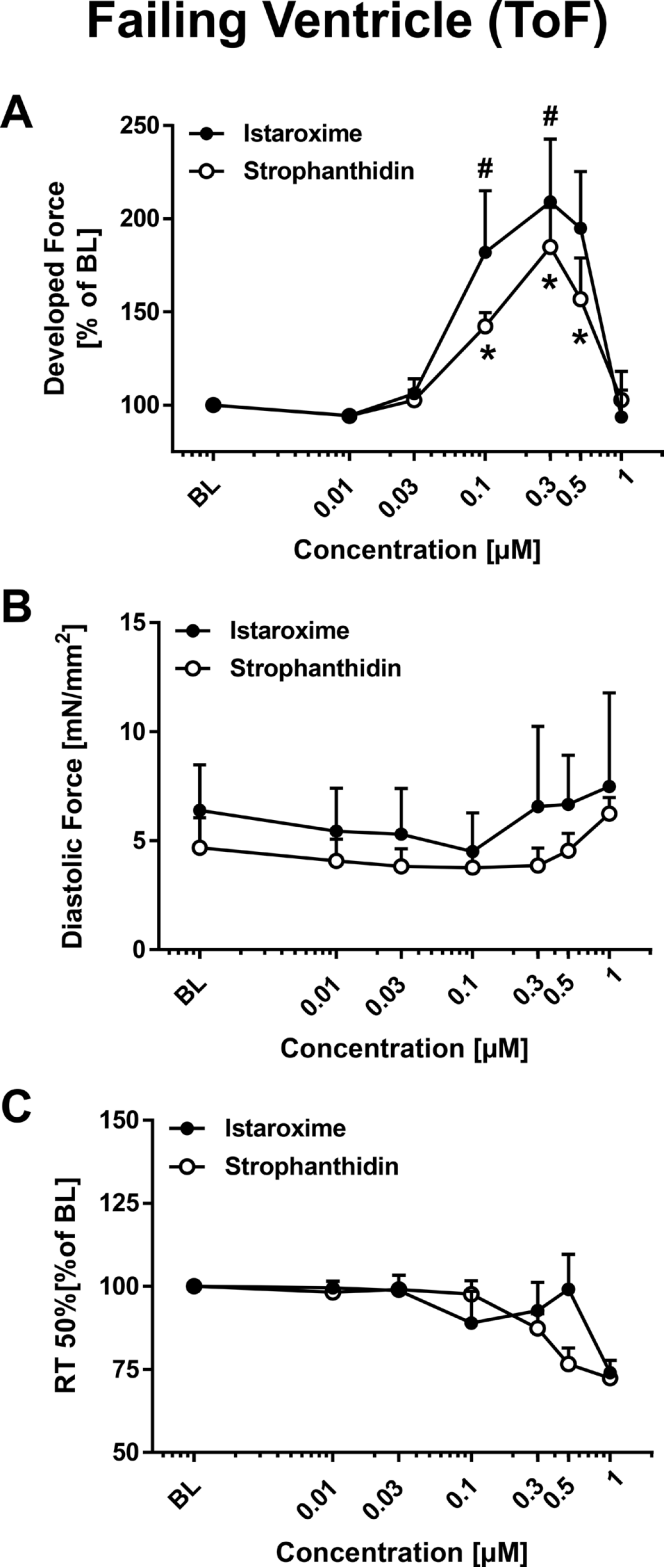
Inotropic effects of istaroxime and strophanthidin on human ventricular myocardium from patients with Tetralogy of Fallot (ToF) Comparable effects of istaroxime and strophanthidin on developed force, diastolic force, and RT50% in ventricular myocardium from infants with Tetralogy of Fallot (ToF). Data are represented as mean +/− SEM. (**A**) Dose response relationship (DRR) for strophanthidin (open circles; *n* = 8 trabeculae) and istaroxime (filled circles; *n* = 11 trabeculae). Effect on developed force: * = *p* < 0.05 vs. baseline (strophanthidin): ^#^ = *p* < 0.05 vs. baseline (istaroxime). (**B**) DRR for strophanthidin (open circles) and istaroxime (filled circles) with effect on diastolic force. (**C**) DRR for strophanthidin (open circles; *n* = 8 trabeculae) and istaroxime (filled circles; *n* = 11 trabeculae) with effect on RT50% (% of baseline).

After testing the effects of istaroxime and strophanthidin in atrial and ventricular nonfailing trabeculae, we had the unique opportunity to measure inotropic response in failing ventricular trabeculae isolated from patients diagnosed with Tetralogy of Fallot (ToF). ToF is the most frequent cyanotic congenital heart defect. The anatomic abnormalities of ToF, such as interventricular communication, subpulmonary stenosis, biventricular origin of the aortic valve, and right ventricular hypertrophy cause right ventricular restrictive physiology and impaired relaxation [24, 25]. Figure 2 shows the effects of istaroxime (*n* = 11 trabeculae) versus strophanthidin (*n* = 8 trabeculae) on developed force (Figure 2A), diastolic force (Figure 2B) and RT50% (Figure 2C). Both substances exhibited dose-dependent positive inotropic effects with a maximal inotropic effect at 0.3 μM. Consistent with our findings from nonfailing atrium and ventricle, continuing to increase the concentration reversed the effects of both substances in the failing myocardium. Diastolic force (Figure 2B) and RT50% (Figure 2C) did not change with istaroxime or strophanthidin administration. An FFR protocol was not performed since the number of acquired trabeculae from the ToF tissue was too little for an experimental series. No arrhythmic events were observed in any of the experiments performed using failing ventricular myocardium.

### Istaroxime exerts stronger maximal positive inotropic effects than strophanthidin in failing myocardium (ToF)

**Figure 3:**
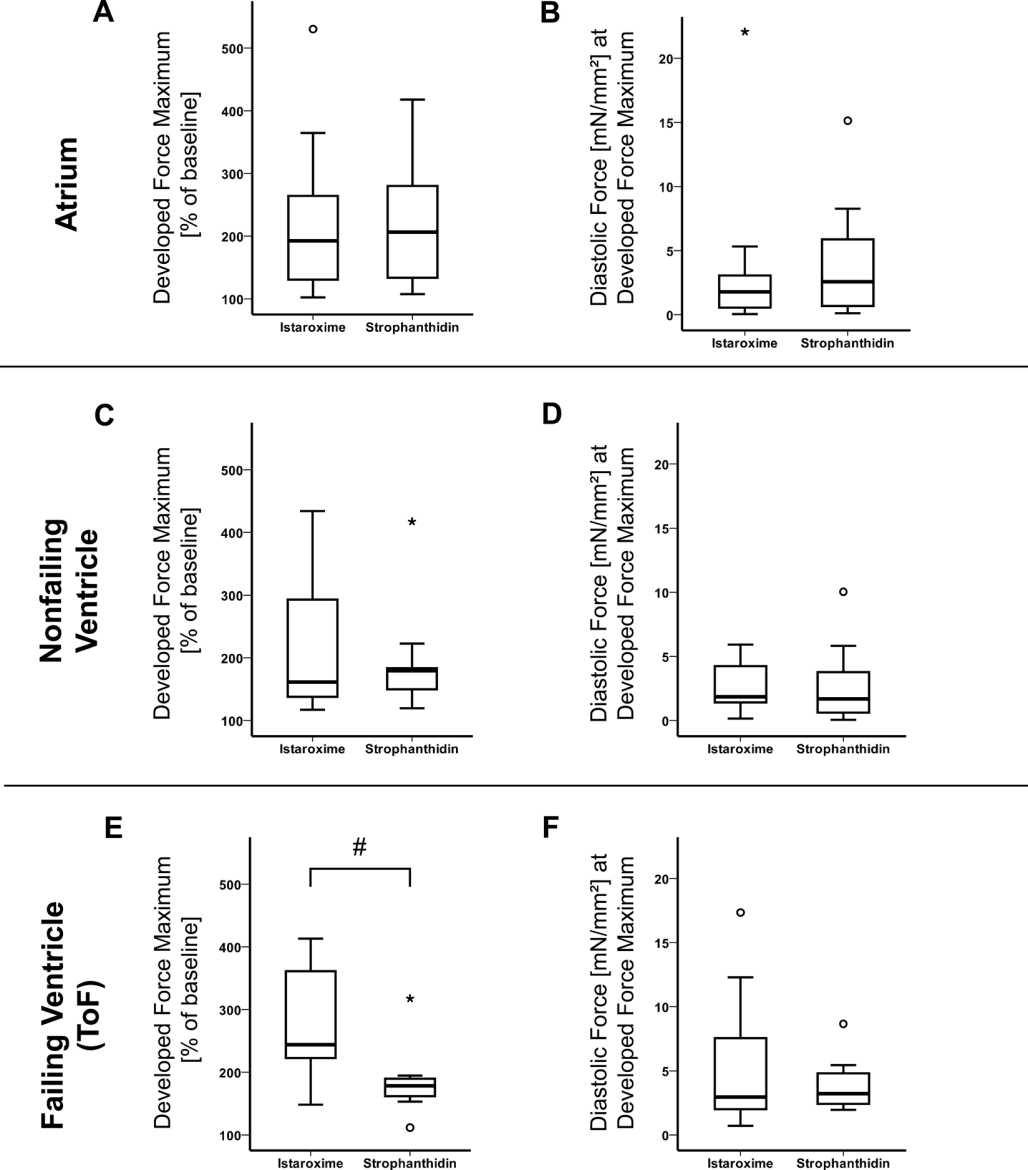
Maximal inotropic effect (istaroxime vs. strophanthidin) Istaroxime enhances maximum developed force in failing trabeculae more than strophanthidin, but not maximum diastolic tension. Data are represented as mean +/− SEM. (**A**) Box plot comparing maximum developed force values of strophanthidin (*n* = 8 trabeculae) and istaroxime (*n* = 13 trabeculae), regardless of the concentration at which maximum developed force was reached, in atrium (% of baseline). (**B**) Box plot comparing diastolic force values (corresponding values to (A) of strophanthidin (*n* = 8 trabeculae) and istaroxime (*n* = 13 trabeculae) in atrium (mN/mm^2^). (**C**) Box plot comparing maximum developed force values of strophanthidin (*n* = 9 trabeculae) and istaroxime (*n* = 9 trabeculae) in nonfailing ventricle (% of baseline). (**D**) Box plot comparing diastolic force values (corresponding values to (C) of strophanthidin (*n* = 9 trabeculae) and istaroxime (*n* = 9 trabeculae), in nonfailing ventricle (mN/mm^2^). (**E**) Box plot comparing maximum developed force values of strophanthidin (*n* = 8 trabeculae) and istaroxime (*n* = 11 trabeculae) in failing ventricle (% of baseline): ^#^ = *p* < 0.05. (**F**) Box plot comparing diastolic force values (corresponding values to (E) of strophanthidin (*n* = 8 trabeculae) and istaroxime (*n* = 11 trabeculae) in failing ventricle (mN/mm^2^). ° = outliers, * = extreme outliers.

In order to better compare the inotropic effects caused by strophanthidin and istaroxime, we used the data from the DRR for atrial, nonfailing ventricular, and failing ventricular trabeculae to focus on the maximal developed force, regardless of the concentration at which the maximum was reached. Figure 3 shows box plots of the maximal developed force and of the corresponding diastolic force, respectively. The analysis revealed a significant difference in the respective influence of istaroxime and strophanthidin on maximal developed force in failing hearts (Figure 3E). The maximal developed force in failing trabeculae was significantly higher in the istaroxime group than in the strophanthidin group (283% ± 26% of baseline vs. 186% ± 21% of baseline, *p* = 0.009). The diastolic force measured at the time of maximal developed force did not differ between istaroxime and strophanthidin (Figure 3F). We did not observe significant differences in the maximal developed force between drugs in neither atrial (Figure 3A–3B) nor nonfailing ventricular (Figure 3C–3D) trabeculae. The same analyses were performed on data obtained from FFR experiments. Increasing stimulation frequencies did not reveal any significant differences between istaroxime and strophanthidin on maximal developed force, diastolic force or RT50% in atrial or nonfailing ventricular myocardium (Supplementary Figure 4A–4F). Thus, istaroxime exerts stronger maximal positive inotropic effects than strophanthidin in failing ventricular myocardium.

## DISCUSSION

Our major findings are: (1) istaroxime has positive inotropic effects in human atrial, human nonfailing ventricular, and human failing ventricular myocardium; (2) istaroxime and strophanthidin show comparable effects with respect to dose response (DRR) and force-frequency relationship (FFR); (3) istaroxime has stronger maximal positive inotropic effects than strophanthidin in failing human myocardium.

### Functional and toxic effects of istaroxime and strophanthidin in human myocardium

The DRR that was performed to compare the effects of strophanthidin and istaroxime on the developed force in human atrial, nonfailing ventricular, and failing ventricular trabeculae (ToF) showed that increased concentrations of istaroxime as well as strophanthidin resulted in a positive inotropic response. Supraphysiological concentrations of istaroxime and strophanthidin exhibited a toxic effect causing a decline in developed force. These results are similar to what has previously been reported on the effects of istaroxime in murine ventricular myocytes, where cell shortening (inotropy) was investigated in a concentration-dependent fashion [26]. Chronic istaroxime has also been shown to improve cardiac function and heart rate variability in hamster cardiomyopathy models [27]. In our study, both istaroxime and strophanthidin slightly increased diastolic force in a dose-dependent manner compared to baseline with comparable curve kinetics in atrial and nonfailing ventricular myocardium. Overall, strophanthidin and istaroxime had similar functional effects in human atrial, nonfailing and failing ventricular myocardium. The similarities in the DRR and FFR were unexpected, especially when considering the mechanistic effects of istaroxime [28], and taking into account the fact that istaroxime is significantly less pro-arrhythmic than digoxin [26]. In our experiments, neither strophanthidin nor istaroxime treatment resulted in any detected arrhythmic events, even at high concentrations of both substances and high Ca^2+^ concentrations (2.5 mM) used in all experiments. This data implicates a comparable safety profile for istaroxime with respect to other NKA inhibitors.

### Effects of istaroxime in an *in-vitro* model of heart failure

We had initially hypothesized that additional SERCA2a stimulation would be beneficial, especially when relaxation is impaired, such as in the conditions of our ToF HF model. Interestingly, SERCA2a stimulation by istaroxime did not reduce diastolic force or accelerate RT50% in any of our tested tissues. However, our analysis revealed that the maximal developed force in failing ventricular trabeculae was significantly higher when treated with istaroxime compared to strophanthidin. This difference did not occur in atrial or in nonfailing ventricular trabeculae. The positive inotropic effect of both compounds is mediated through NKA inhibition, with subsequent increase of intracellular Ca^2+^. Our findings in atrial and nonfailing ventricular myocardium suggest istaroxime and strophanthidin inhibit the NKA to a similar extent. The exact extent of NKA inhibition by istaroxime compared to strophanthidin has never been quantified. Since ToF is a condition with impaired relaxation and restrictive right ventricular physiology [25, 29], we can only speculate that the additional SERCA2a stimulation by istaroxime in a setting of impaired relaxation is responsible for a more pronounced increase in maximal developed force. We conclude that in our in-vitro model of HF, istaroxime has stronger maximal positive inotropic effects than strophanthidin. This is in line with the results of experiments using a canine model of acute myocardial infarction where istaroxime had a greater effect on contractility than dobutamine and also improved relaxation, while dobutamine did not [30].

Our *in-vitro* DRR findings further complement the findings from the HORIZON-HF trial, where istaroxime was tested in acute heart failure syndromes using a comprehensive assessment of cardiovascular function in addition to hemodynamic measurements [31]. Other studies performed in dogs with advanced heart failure showed istaroxime significantly increased LV ejection fraction in a dose-dependent manner. In addition, istaroxime significantly reduced LV end-diastolic pressure and end-diastolic wall stress [15]. The latter effect contradicts with our findings. Another phase 1–2 dose-escalating study evaluating the safety and tolerability of istaroxime suggested that istaroxime is potentially useful in the treatment of HF [32]. We found only one report on the effects of istaroxime using myocardial trabeculae from patients undergoing cardiac transplantation [33]. In that study, istaroxime (100 nM - 1.0 μM) increased developed tension and twitch kinetics in a dose-dependent manner while stimulating SERCA2a activity in sarcoplasmic reticulum microsomes at physiological free calcium concentrations. Istaroxime is currently the only compound available that stimulates SERCA2a activity and produces a luso-inotropic effect in HF [33].

### NKA inhibition in heart failure and potential link to prostate cancer

Classical inotropic agents, such as beta1-adrenergic agonists, phosphodiesterase III inhibitors and Ca^2+^ sensitizers improve HF symptoms and hemodynamics by increasing the levels of free intracellular Ca^2+^ and thus contractility, but also increase myocardial oxygen demand and exert arrhythmogenic effects [34]. Despite the acute hemodynamic benefits, these inotropes play no role in the management of chronic advanced HF due to inherent adverse side-effect profiles [11, 15, 35]. Instead, a very well established protocol of therapeutic escalation levels is given in the European Society of Cardiology (ESC) heart failure guidelines [36]. NKA inhibitors may provide additional help in treatment with a class of recommendation IIb and a level of evidence B. This low class of recommendation is due to the potential harmful effects of digoxin for women [37] and controversial reported effects in meta-analysis for both digoxin and digitoxin [38–40]. In summary, NKA inhibition does not lower mortality but does seem to lower the incidence of decompensation of heart failure and the hospitalization rate for heart failure. Of note, istaroxime is not currently featured as an established approach in the ESC guidelines for heart failure. However, the reported negative effects for women caused by the NKA inhibitor digoxin are not an issue in terms of its use as a PC treatment. Keeping in mind that HF can develop as a side effect of hormonal therapy in PC, the additional use of inotropes could be beneficial. Moreover, istaroxime itself exerts PC inhibiting effects. The median age of PC patients at time of diagnosis is 66 years. HF patients, especially those diagnosed with Heart Failure with preserved Ejection Fraction (HFpEF), are also usually at an advanced age [41]. HFpEF accounts for about 50% of all cases of HF [42–44] and its prevalence relative to Heart Failure with reduced Ejection Fraction (HFrEF) is growing by 10% per decade [44]. The vast majority of published reports assessing the effects of istaroxime focused on preclinical HFrEF models and HFrEF patients. We tested istaroxime in a novel in-vitro HF model with diastolic impairment, which is a hallmark of HFpEF, and demonstrated that istaroxime exerts stronger maximal inotropic effects compared to strophanthidin.

The presented information suggests that istaroxime has the potential to be a beneficial add-on therapy for PC, exerting direct anti-cancer effects and counteracting potential side effects of the basic therapy with respect to toxic effects at supraphysiological concentrations.

### Limitations and strengths of the study

Compared to established animal models that mimic heart failure, our HF model is not a classic experimental model of heart failure with preserved or reduced ejection fraction. However, the use of human tissue for all experiments enhances the translational nature of our findings. The study is clearly limited by the small number of ToF patients as a source of failing ventricular trabeculae, resulting in a rather small number of experiments.

We did not directly dissect the inhibitory effect on the NKA from the stimulatory effect on SERCA2a. However via comparison to the NKA inhibitor strophanthidin, which has no known SERCA2a stimulatory effect, our results suggest that SERCA2a stimulation plays a role. To the best of our knowledge, no study has attempted to determine which SERCA isoform is primarily expressed in ventricular ToF myocardium. This is relevant since istaroxime is effective on the SERCA2a isoform in the presence of phospholamban, but not on the SERCA1 alone [45].

On the other hand, the strength of this study is that it was performed in human tissue and carefully assessed the effects of istaroxime and strophanthidin in atrial, nonfailing, and failing ventricular myocardium. Our HF model is of high value, yet no single model can entirely reflect the complexity of heart failure as a clinical syndrome.

## MATERIALS AND METHODS

We assessed three different types of human cardiac tissue: right atrial appendages, nonfailing left ventricles, and right ventricles from infants diagnosed with Tetralogy of Fallot (ToF). The ToF tissue served as a heart failure model.

### Human myocardium and trabeculae preparation

Right atrial appendages were obtained from hearts of patients (*n* = 22 patients) undergoing cardiac surgery for aortocoronary bypass or valve replacement. Left ventricular trabeculae were obtained from human nonfailing donor hearts (*n* = 8 hearts) that were not suitable for transplantation. ToF right ventricular tissue, which was considered the failing sample, was obtained during the surgical reconstruction procedure of the right ventricular outflow tract (*n* = 9 patients). The study was approved by the local ethics committee and patients gave informed written consent. Endocardial trabeculae were prepared in cardioprotective solution, as described in the supplement.

### Experimental protocol

Trabeculae were gradually stretched to the length at which maximal force development was achieved (baseline). After recording a steady state at baseline, we performed two different protocols, a dose response relationship (DRR) and a force frequency relationship (FFR). For the DRR, trabeculae were exposed to increasing concentrations of either istaroxime or strophanthidin (10 nM to 1 μM). After 30 minutes of incubation and reaching steady-state conditions, developed force, diastolic force, and RT50% were recorded. Then the next concentration step was performed. Moreover, we performed FFR by increasing stimulation frequency stepwise from 0.5 Hz to 3.0 Hz. Upon reaching a steady state, kinetic parameters were recorded, and FFR was repeated in the presence of 0.1 μM istaroxime or strophanthidin.

### Drugs

Istaroxime was provided by SIGMA-TAU Industrie Farmaceutiche Riunite S.p.A., Rome – Italy and strophanthidin was purchased from Calbiochem.

### Statistical analysis

For each separate group (atrium, nonfailing ventricle, failing ventricle), we performed a repeated measures analysis of variance (rmANOVA) with all data rank transformed. *P*-values for contrasts were corrected for multiple comparisons according to Bonferroni. Differences in maximum were assessed with the Wilcoxon rank sum test. *P-value*s less than 0.05 were considered statistically significant. All computations were done using IBM SPSS Statistics (Release 21.0.0.0 2012. Armonk, NY, USA). Detailed statistical analysis is provided in the supplement.

## CONCLUSIONS

This is the first report of the functional effects of istaroxime on human myocardium under conditions similar to HF. We found close similarities between istaroxime and strophanthidin in their effect on systolic force, diastolic force, and relaxation time in nonfailing tissue. However, using ToF as a model of heart failure, istaroxime showed stronger maximal positive inotropic effects than strophanthidin. Combining our *in-vitro* results with potential PC inhibiting effects, istaroxime may provide a new therapeutic option for conservative management of PC that counteracts harmful side effects of current therapy strategies and merits further investigation of its clinical use.

## SUPPLEMENTARY MATERIALS FIGURES



## Abbreviations

DRR: dose-response relationship
ESC: European Society of Cardiology
FFR: force-frequency relationship
HF: heart failure
NKA: Na^+^-K^+^-ATPase
PC: prostate cancer
RT50%: time from contraction peak to 50% relaxation
SEM: standard error of the mean
SERCA2a: sarco/endoplasmic reticulum Ca^2+^-ATPase 2a
ToF: Tetralogy of Fallot.
