# Supplementary material for: Istaroxime, a potential anticancer drug in prostate cancer, exerts beneficial functional effects in healthy and diseased human myocardium
Source: Oncotarget. 2017 Apr 30;8(30):49264–74. doi: 10.18632/oncotarget.17540 (PMC5564766; doi:10.18632/oncotarget.17540)
Supplement: Supplementary file 1 [file oncotarget-08-49264-s001.pdf]

Istaroxime, a potential anticancer drug in prostate cancer, exerts beneficial functional effects in healthy and diseased human myocardium

SUPPLEMENTARY MATERIALS

Force frequency relationship-atrium

Using different pacing frequencies, we investigated changes in developed and diastolic force in response to strophanthidin (Supplementary Figure 1A–1B; *n* = 17 trabeculae) and istaroxime (Supplementary Figure 1C–1D; *n* = 19 trabeculae), respectively (force-frequency relationship, 0.5 to 3 Hz; FFR). The same was true for istaroxime (0.1 µM). Moreover, with respect to diastolic force, both substances showed no difference versus baseline in the force frequency relationship. Supplementary Figure 3 shows the effect of both substances (in increasing concentrations) on the RT50% (= time from peak tension to 50% relaxation) (A) and the effect of each substance at 0.1 µM is further investigated versus baseline using different pacing frequencies (B and C). Overall, strophanthidin and istaroxime reveal similar functional effects in human atrial myocardium.

Force frequency relationship – non-failing ventricle

Using different pacing frequencies, we further investigated changes in developed and diastolic force in response to strophanthidin (Supplementary Figure 2A–2B; *n* = 7 trabeculae) and istaroxime (Supplementary Figure 2C–2D; *n* = 7 trabeculae), respectively (FFR, 0.5 to 3 Hz). Strophanthidin (0.1 µM; Supplementary Figure 2A) and istaroxime (0.1 µM; Supplementary Figure 2C) exerted noticeable similarities with respect to their force frequency relationship. With respect to diastolic force, both substances showed no significant difference versus baseline in the force frequency relationship (Supplementary Figure 2B and 2D). Supplementary Figure 3D shows changes of RT50% with administration of either substance in increasing concentrations. Further investigation of each substance at 0.1µM using different pacing frequencies (Supplementary Figure 3E–3F) revealed no significant difference compared to baseline. Thus, strophanthidin and istaroxime reveal similar functional effects in human ventricular myocardium.

Comprehensive force frequency relationship analysis

In order to identify potential differences between strophanthidin and istaroxime, we took a closer look focusing only on the maximum developed force values presented in the previous FFR figures (S1/S2). We analyzed the maximal developed force recorded after incubation with 0.1 µM istaroxime and 0.1 µM strophanthidin, respectively, regardless of the pacing frequency. Supplementary Figure 4 shows box plots of the maximal developed force, and the corresponding diastolic force and RT50% values, irrespective of the pacing frequency. This representation allows for a better comparison between strophanthidin and istaroxime, and for detection of differences that might otherwise be overlooked. However, analysis of both atrial (Supplementary Figure 4A–4C) and ventricular (Supplementary Figure 4D–4F) preparations revealed no significant difference in the influence of istaroxime and strophanthidin on FFR.

Trabeculae preparation

With the help of a stereomicroscope, trabeculae were carefully dissected, transferred to an organ bath and fixed on hooks, stretched to optimal length in tyrode's solution with a Ca^{2+} concentration of 2.5 mM and electrically stimulated. Trabeculae were maintained at 37°C. Developed force, diastolic force and RT50% were recorded using a force-transducer (Scientific Instruments, Germany). Tracings were recorded on a thermorecorder and stored digitally for offline analysis.

Statistical analysis

For each separate group (atrium, nonfailing ventricle, failing ventricle (ToF)) we performed a repeated measures analysis of variances (rmANOVA) with all data rank transformed to test the effects on the muscle force parameters of the concentration level (baseline, 0.01, 0.03, 0.1, 0.3, 0.5, 1) as within subjects factor and the two groups (istaroxime and strophanthidin) as between subjects factor. We defined contrasts to test which

concentration level differs significantly from baseline in total and within each group. Beyond the main between subject effect of a group difference, we defined contrasts to test at which concentration level the two groups differ significantly and whether there is a significant interaction of group*concentration between two adjacent concentration levels. *P*-values for contrasts were corrected for multiple comparisons according to Bonferroni. Differences in maximum developed force and differences in diastolic force and RT50% at the concentration level at which the muscle strips attained maximum developed force were assessed with the Wilcoxon rank sum test. *P*-values less than 0.05 were considered statistically significant. All computations were done using IBM SPSS Statistics (Release 21.0.0.0 2012. Armonk (NY), USA: International Business Machines Corporation).

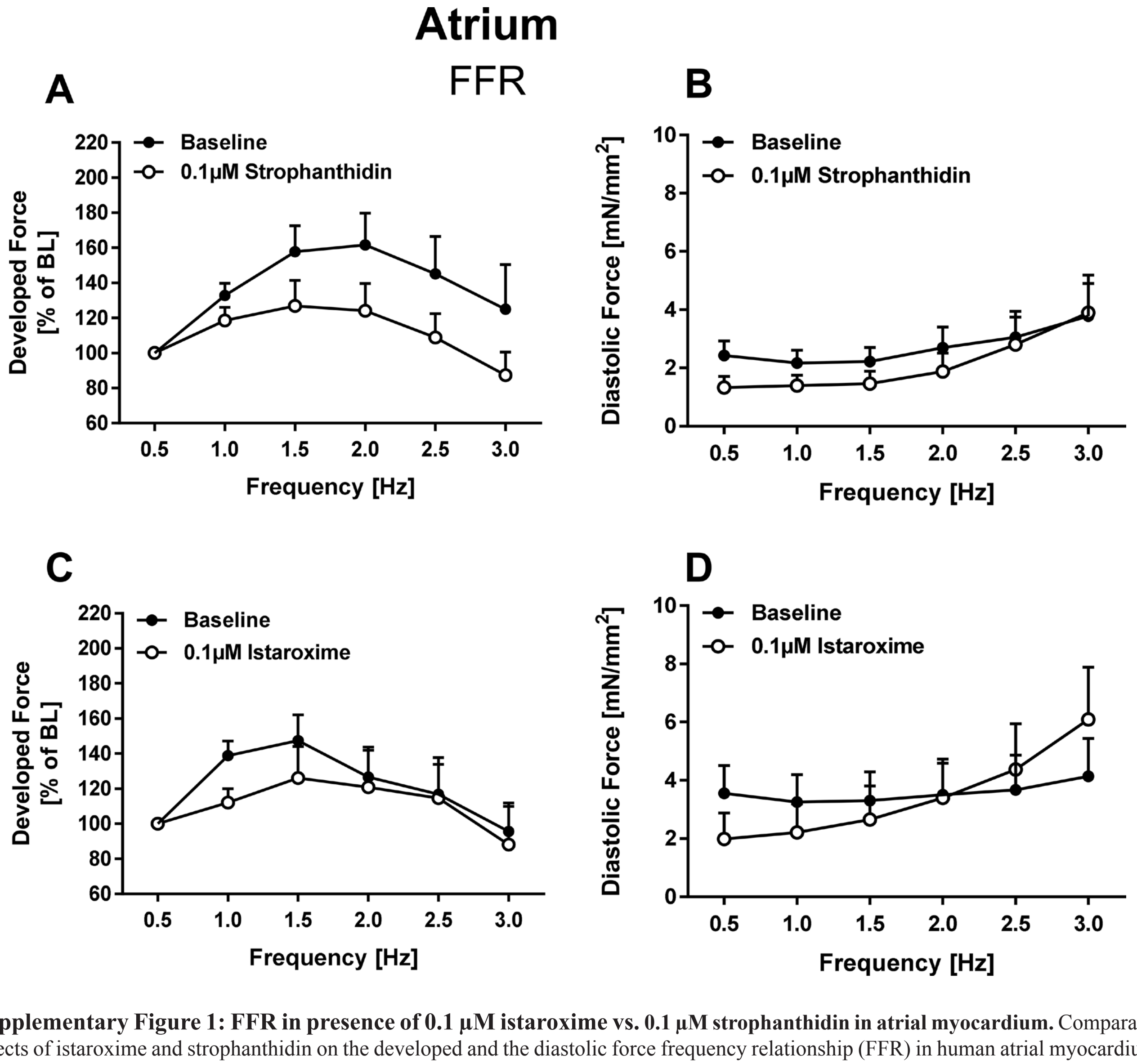

Supplementary Figure 1: FFR in presence of 0.1 µM istaroxime vs. 0.1 µM strophanthidin in atrial myocardium. Comparable effects of istaroxime and strophanthidin on the developed and the diastolic force frequency relationship (FFR) in human atrial myocardium. Data are represented as mean +/− SEM. (**A**) Developed FFR in the presence of 0.1 µM strophanthidin (open circles; n = 17 trabeculae) vs. baseline (filled circles). (**B**) Diastolic FFR in the presence of 0.1 µM strophanthidin (open circles; n = 17 trabeculae) vs. baseline (filled circles). (**C**) Developed FFR in the presence of 0.1 µM istaroxime (open circles; n = 19 trabeculae) vs. baseline (filled circles). (**D**) Diastolic FFR in the presence of 0.1 µM istaroxime (open circles; n = 19 trabeculae) vs. baseline (filled circles).

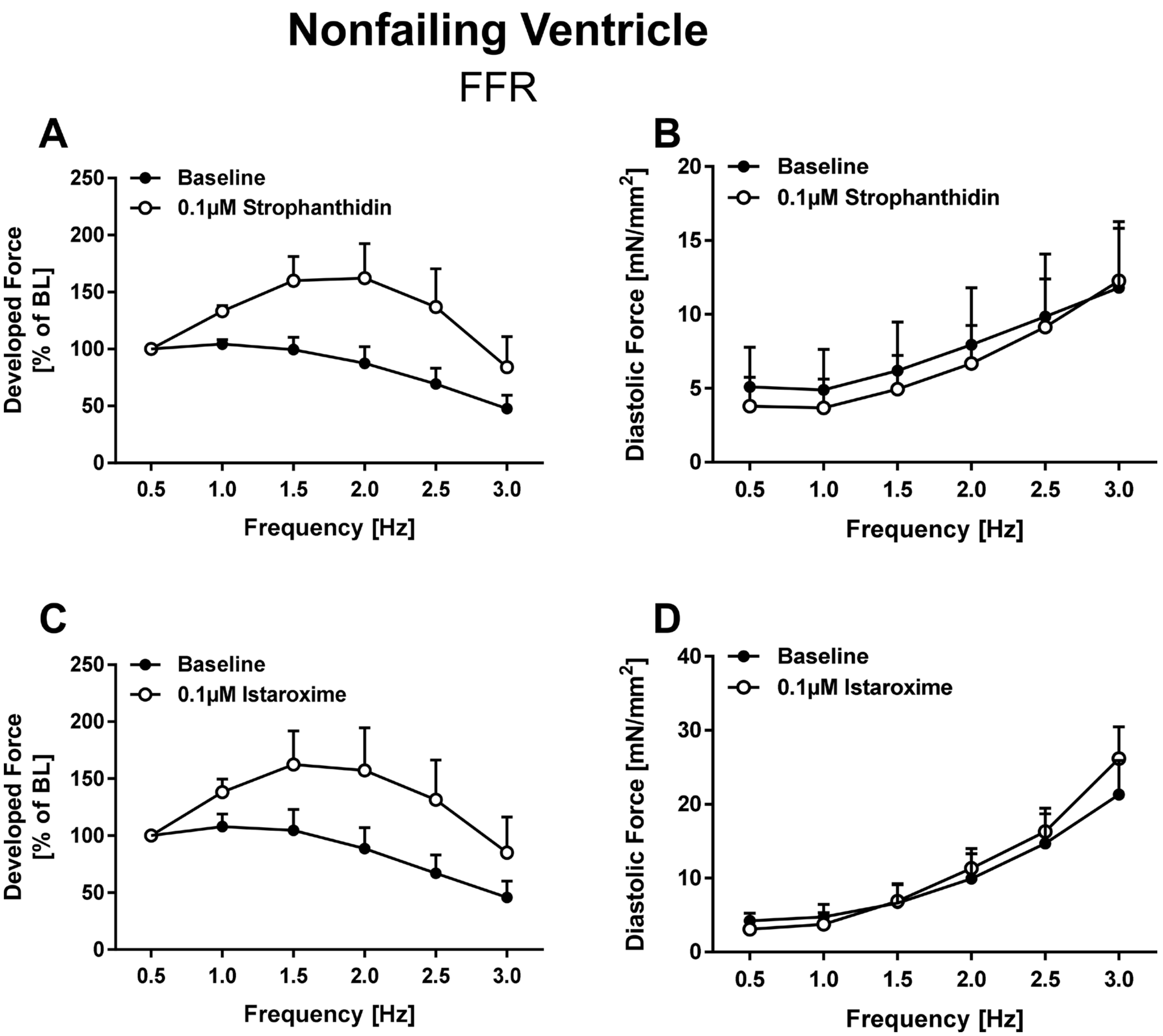

Supplementary Figure 2: FFR in presence of 0.1 µM istaroxime vs. 0.1 µM strophanthidin in nonfailing ventricular myocardium. Comparable effects of istaroxime and strophanthidin on the developed and the diastolic force frequency relationship (FFR) in human nonfailing ventricular myocardium. Data are represented as mean +/− SEM. (**A**) Developed FFR in the presence of 0.1 µM strophanthidin (open circles; *n* = 7 trabeculae) vs. baseline (filled circles). (**B**) Diastolic FFR in the presence of 0.1 µM strophanthidin (open circles; *n* = 7 trabeculae) vs. baseline (filled circles). (**C**) Developed FFR in the presence of 0.1 µM istaroxime (open circles; *n* = 7 trabeculae) vs. baseline (filled circles). (**D**) Diastolic FFR in the presence of 0.1 µM istaroxime (open circles; ; *n* = 7 trabeculae) vs. baseline (filled circles).

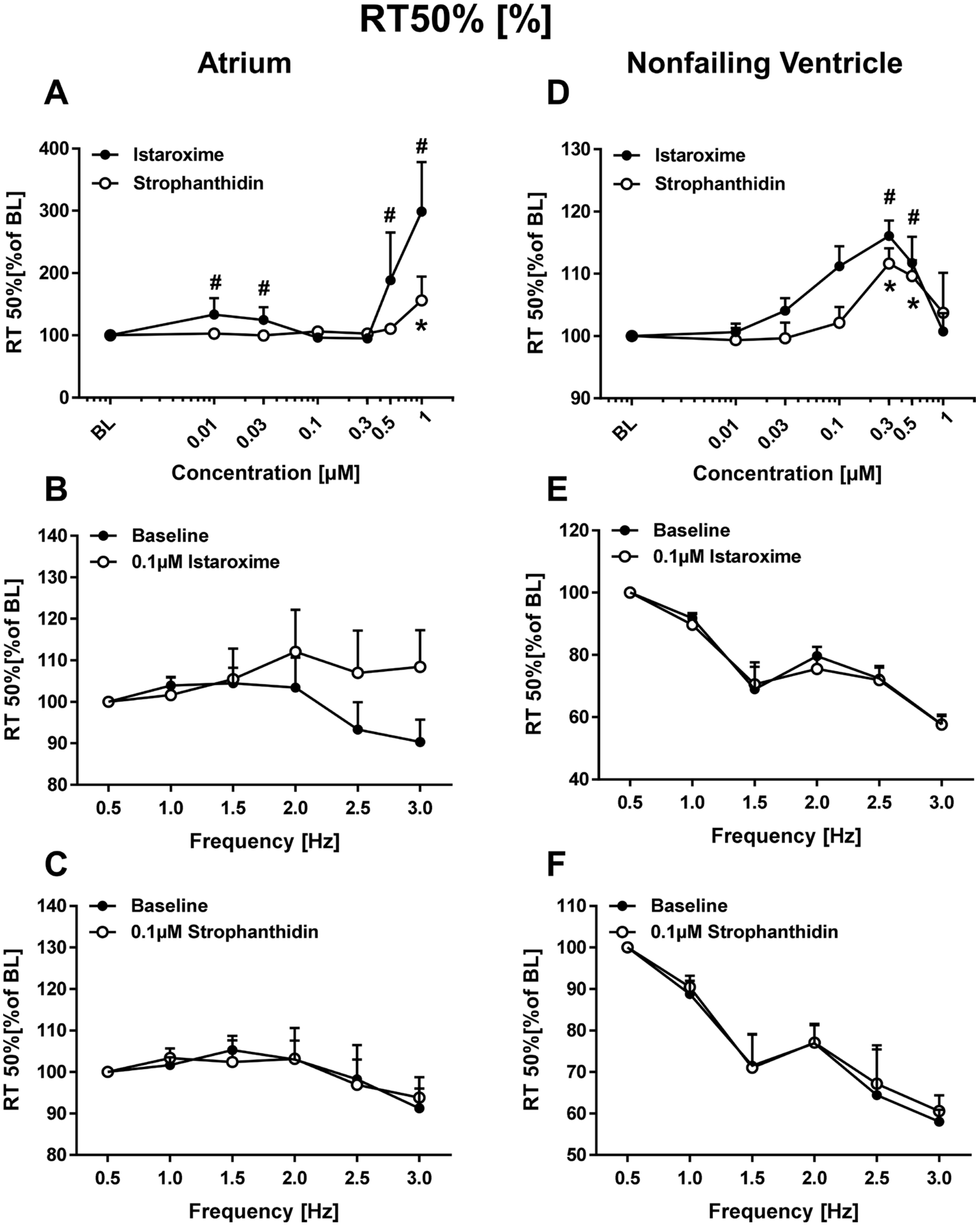

Supplementary Figure 3: Analysis of RT50%. Comparable effects of istaroxime and strophanthidin on the time from contraction peak to 50% relaxation (RT50%) in human atrial (**A**, **B**, D) and nonfailing ventricular (**D**, **E**, **F**) myocardium. Data are represented as mean +/− SEM. (A) Dose response relationship (DRR) for strophanthidin (open circles; n = 13 trabeculae) and istaroxime (filled circles; n = 8 trabeculae) in atrial myocardium. Effect on RT50%; * = $p < 0.05$ vs. baseline (strophanthidin); # = $p < 0.05$ vs. baseline (istaroxime). (B) Atrial force frequency relationship (FFR) on RT50% in the presence of 0.1 µM istaroxime (open circles; n = 19 trabeculae) vs. baseline (filled circles). (**C**) Atrial FFR on RT50% in the presence of 0.1 µM strophanthidin (open circles; n = 17 trabeculae) vs. baseline (filled circles). (D) DRR for strophanthidin (open circles; n = 9 trabeculae) and istaroxime (filled circles; n = 9 trabeculae) in nonfailing ventricular myocardium. Effect on RT50%; * = $p < 0.05$ vs. baseline (strophanthidin); # = $p < 0.05$ vs. baseline (istaroxime). (E) Ventricular FFR on RT50% in the presence of 0.1 µM istaroxime (open circles; n = 7 trabeculae) vs. baseline (filled circles). (F) Ventricular FFR on RT50% in the presence of 0.1 µM strophanthidin (open circles; n = 7 trabeculae) vs. baseline (filled circles).

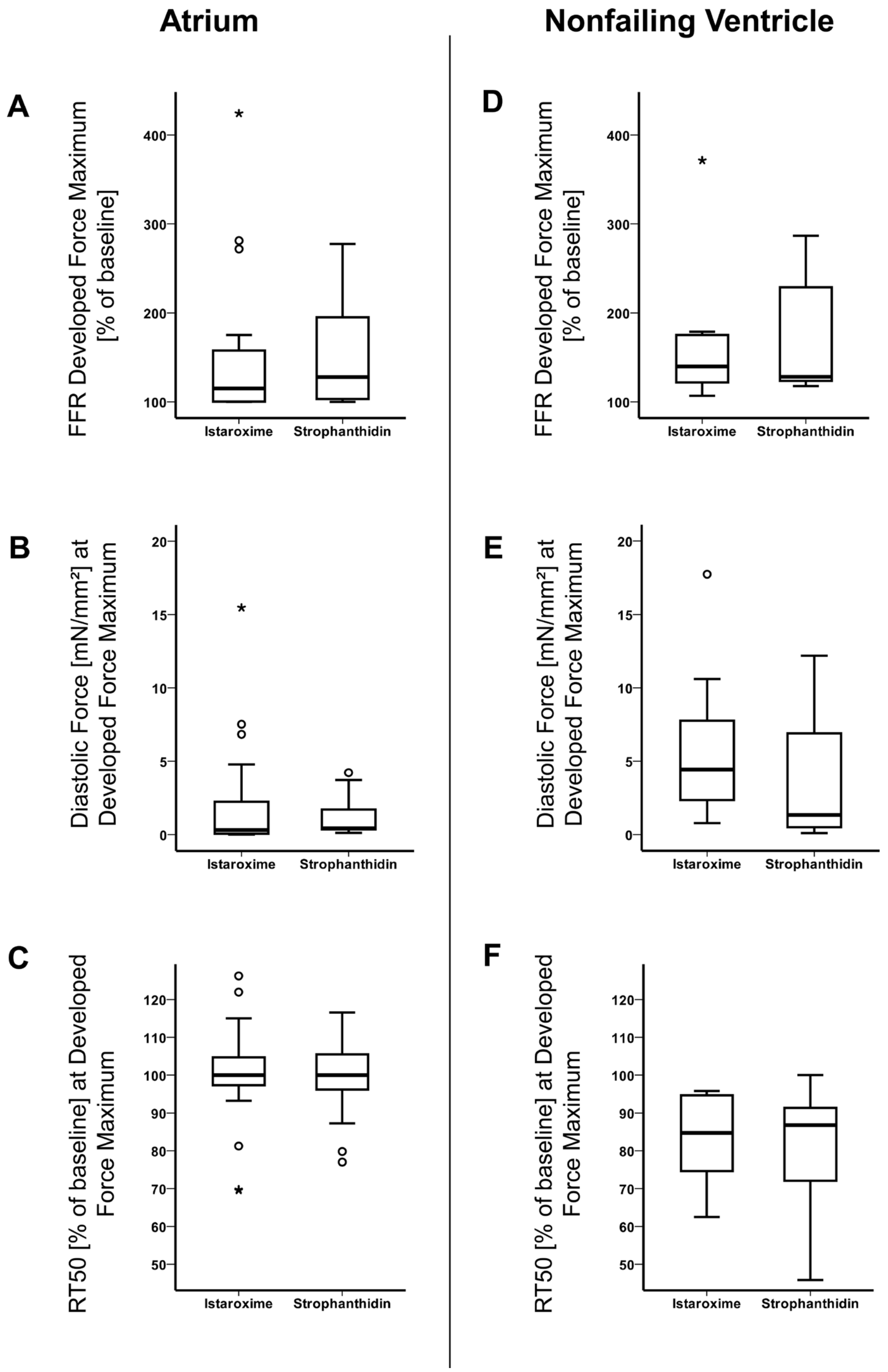

Supplementary Figure 4: Maximal developed and diastolic force in the FFR protocol. Istaroxime (0.1 µM) affects FFR maximum values similarly to strophanthidin (0.1 µM) in atrial and ventricular myocardium, regardless of the stimulation frequency at which the maximum was reached. Data are represented as mean +/− SEM. (**A**) Box plot comparing maximum force frequency relationship (FFR) values of strophanthidin (*n* = 17 trabeculae) and istaroxime (*n* = 19 trabeculae), respectively, with regard to developed force in atrium (in % of baseline). (**B**) Box plot comparing maximum FFR values of strophanthidin (*n* = 17 trabeculae) and istaroxime (*n* = 19 trabeculae) with regard to diastolic force in atrium (mN/mm²). (**C**) Box plot comparing maximum FFR values of strophanthidin (*n* = 17 trabeculae) and istaroxime (*n* = 19 trabeculae) with regard to RT50% in atrium (% of baseline). (**D**) Box plot comparing maximum FFR values of strophanthidin (*n* = 7 trabeculae) and istaroxime (*n* = 7 trabeculae) with regard to developed force in ventricular myocardium (% of baseline). (**E**) Box plot comparing maximum FFR values of strophanthidin (*n* = 7 trabeculae) and istaroxime (*n* = 7 trabeculae) with regard to diastolic force in ventricular myocardium (mN/mm²). (**F**) Box plot comparing maximum FFR values of strophanthidin (*n* = 7 trabeculae) and istaroxime (*n* = 7 trabeculae) with regard to RT50% in ventricular myocardium (% of baseline). ° = outliers, * = extreme outliers.
